# Fatty acid-binding protein 5 deficiency impairs alveolar macrophage function and metabolism

**DOI:** 10.1016/j.jlr.2026.101069

**Published:** 2026-06-23

**Authors:** Jack H. Ratliff, Katja Aviszus, Sophia Addi, Manale El Kharbili, Steven Fujaros, Sarah K. Sasse, Brian E. Vestal, Anthony N. Gerber, Evgenia Dobrinskikh, Fabienne Gally

**Affiliations:** 1 Department of Immunology and Genomic Medicine, National Jewish Health, Denver, Colorado, USA; 2 Department of Medicine, National Jewish Health, Denver, Colorado, USA; 3 Department of Medicine, University of Kentucky, Lexington, Kentucky, USA; 4 Department of Medicine, University of Colorado, Aurora, Colorado, USA; 5 Department of Pediatrics, University of Colorado, Aurora, Colorado, USA; 6 Department of Immunology and Microbiology, University of Colorado, Aurora, Colorado, USA

**Keywords:** COPD, resolution of inflammation

## Abstract

Macrophages are large mononuclear immune cells that participate in host protection, not only by phagocytosing foreign or infected cells and initiating inflammatory responses, but also by contributing to the resolution of inflammation. Chronic Obstructive Pulmonary Disease (COPD) is characterized by increased numbers of macrophages in lung tissue, with altered engulfment capabilities. However, the molecular pathways leading to macrophage dysfunction in COPD remain unclear. Using integrated genetics and genomics approaches, we previously identified Fatty Acid Binding Protein 5 (FABP5) as a key target in the resolution of airway inflammation that exhibits decreased expression in COPD patients. The objective is to define the significance of alveolar macrophage FABP5 by comparing the resolution of inflammation in WT and Fabp5^−/−^ mice following nontypeable *Haemophilus influenzae* (*NTHi*) infection or LPS sterile inflammation. Immune and metabolic responses were analyzed using functional assays, flow cytometry, ELISA, cell metabolic profiling and tracing, as well as ATAC-seq. Fabp5^−/−^ mice exhibited impaired efferocytosis, reflected by a reduction of apoptotic cell engulfment by alveolar macrophages. This was accompanied by a reduction in fatty acid uptake and fatty acid β-oxidation, a reduction in mitochondrial respiration, an accumulation of TCA cycle and glycolysis metabolites, and an increased chromatin accessibility for AP-1 family members. Fabp5-deficient alveolar macrophages failed to initiate reparative metabolic programming, which is critical for the resolution of inflammation. Our data suggest that increasing FABP5 expression could provide a metabolic switch that facilitates macrophage conversion to a pro-resolving phenotype and restores alveolar macrophage efferocytic functions in the lungs of COPD patients.

The lung microenvironment dictates the phenotype, metabolic state and transcriptome of macrophages, particularly in response to inflammation (1), differentiation (2) and localization (3). Thus, it is understandable that chronic lung diseases, such as Chronic Obstructive Pulmonary Disease (COPD), may alter the phenotype and function of lung macrophages. In COPD, macrophages have been shown to produce higher amounts of inflammatory cytokines and chemokines, reactive oxygen species (ROS) and elastolytic enzymes leading to the recruitment of cytotoxic T cells and neutrophils. In turn, those cells, ROS and enzymes contribute to the destruction of the alveoli, a process known as emphysema (4). Furthermore, the phagocytic capabilities of macrophages are impaired in COPD (5, 6), which may explain why patients have a higher risk of bacterial and/or viral infections (7). Exacerbations, mainly caused by airway infections, have been linked to the development and progression of COPD (8). COPD exacerbations are one of the contributing risk factors for having more exacerbations, suggesting that a vicious cycle of ineffective resolution of inflammation predisposes individuals for future episodes (9, 10). Nontypeable *Haemophilus influenzae* (*NTHi*), a gram-negative bacterium, is one of the most common pathogen found in patients with exacerbations (11).

Our previous studies demonstrated that Fatty Acid Binding Protein 5 (FABP5) is increased during respiratory infections but decreased in COPD (12) and by cigarette smoke (13) through inhibition of c-Jun binding to the FABP5 promoter (14). We have also shown that when FABP5 is decreased, bacterial infections cause more inflammation; in turn, when FABP5 is overexpressed, cells are protected against inflammation via increased peroxisome proliferator-activated receptor γ (PPARγ) activity (15). Furthermore, Fabp5^−/−^ mice undergo severe inflammation following influenza A infection that persists long after wild-type (WT) mice recovery (16), suggesting that Fabp5 may be involved in the resolution of inflammation. We also characterized a novel single-nucleotide polymorphism within the *FABP5* locus that resulted in reduced FABP5 expression and was associated with COPD exacerbations (17). FABP5 belongs to the FABP family of small, highly conserved cytoplasmic proteins that bind long-chain fatty acids and other hydrophobic ligands and are involved in fatty acid uptake, transport, and metabolism (18). FABP5 is highly expressed in alveolar macrophages (19, 20) and plays a critical role in macrophage metabolism and pro-resolving phenotype by interacting with the nuclear receptor PPARγ (21). However, whether the reduction or absence of FABP5 in the different lung macrophages impacts their function or metabolism remains unknown. This is of particular importance given that macrophages are at the center of the processes required for the resolution of inflammation, including engulfment of apoptotic cells, activation of PPARγ, and repair processes (5, 22).

Here, using two different models of resolution of inflammation with different severity and kinetics, namely a live bacteria *NTHi* or sterile inflammation with lipopolysaccharide (LPS) from *Pseudomonas aeruginosa*, we show that alveolar macrophages lacking Fabp5 present impaired apoptotic cell engulfment. This is accompanied by changes in fatty acid metabolism and mitochondrial functions. Given that FABP5 is decreased in COPD (12), this work provides an understanding of the mechanisms by which decreased FABP5 leads to over-inflamed lung tissue in COPD.

## Materials and methods

A complete description of materials and methods can be found in the supplementary file.

### Animals

Fabp5^−/−^ mice and littermate wild-type (WT) controls, on a C57BL/6J background, were kindly provided by Dr Gokhan Hotamisligil and bred in the Biological Resources Center at National Jewish Health. Mice were kept on a 12-h light-dark cycle with *ad libitum* access to food and water. All experimental animals used in this study were covered under protocols approved by the Institutional Animal Care and Use Committee of National Jewish Health.

### *NTHi* infection or LPS sterile inflammation

Eight-to twelve-week-old WT and Fabp5^−/−^ mice were anesthetized by isoflurane (3%–5%) and then inoculated by oropharyngeal aspiration with 50 μl of 1 × 10^6^ colony-forming units (CFU) of *NTHi* or 15 μg of LPS from *Pseudomonas aeruginosa*. As a control, mice were inoculated with 50 μl of saline. Three days post-*NTHi* infection or 9-days post-LPS sterile inflammation, mice were sacrificed with an injection of Fatal Plus (2 μl/g of body weight) to examine lung and bronchoalveolar lavage (BAL) cell profiles for gene expression, histopathology and metabolomic assays.

### Single-cell transcriptome profiling by RNA sequencing and data analysis

Lungs were digested using Liberase TM (0.25 mg/ml, Roche) and DNase (125 μg/ml, Worthington). CD45+ cells were enriched using magnetic beads per manufacturer’s instructions (StemCell Technologies). Single-cell RNA-seq reads were aligned and quantified using Cell Ranger (10X Genomics) with mm10 as the reference genome. Subsequently, filtering was applied to each sample using the Seurat R (23) package where only cells with at least 200 genes detected, at least 1% of the reads aligned to ribosomal genes, and less than 30% of the reads aligned to mitochondrial genes were retained for further analysis. Subsequently, RNA counts were normalized using the SCTransform (24) method available from the eponymous R package, and then the samples were integrated within Seurat using the 3,000 most variable genes as the integration features. Next, principal components analysis (PCA) was done, cells were clustered using the first 30 PCs with a resolution of 0.35, and then Uniform Manifold Approximation and Projection (UMAP) embeddings were calculated for visualizations. For the resulting cell clusters, upregulated marker genes were identified using the Wilcoxon test available in Seurat focusing on genes that were expressed in at least 50% of the cells within the cluster of interest. These lists were used to manually annotate clusters into biologically meaningful cell types that were used for the remainder of the analyses. For differential expression analyses, we applied negative binomial generalized linear models via the edgeR (25) package to gene expression across the individual cells within each cell type independently. Within a given cell type, genes were filtered to only those that had at least as many cells with a counts per million value greater than one as there were cells in the smallest experimental group. From the edgeR model fits, log fold changes and false discovery rates (FDR) (26) were extracted to generate lists of differentially expressed genes.

Identification of enriched pathways in the Kyoto Encyclopedia of Genes and Genomes (KEGG) and Gene Ontology was done using ShinyGO 0.85 (27). Differentially expressed genes were selected for submission based on a *P*-value <0.05. Enriched pathways were selected based on an FDR < 0.05. Volcano plots were generated using VolcaNoseR (28). Heatmaps of differentially expressed genes were generated according to KEGG pathways (29).

### In vivo apoptotic cell clearance

Murine thymocytes were isolated from 4-6-week-old C57BL/6J mice. Individual cells were obtained by passing thymi through a 40-μm cell strainer. Thymocytes were washed with PBS, resuspended in RPMI media containing 10% FBS at 2 × 10^6^ cells/ml, exposed to UV irradiation for 10 min, and incubated for 3 h in 5% CO_2_ at 37°C. Cells were counted, spun at 1,000 rpm for 5 min and resuspended in PBS at 200 × 10^6^ cells/ml. Recipient mice, either WT or Fabp5^−/−^, were anesthetized with isoflurane (4%) and 50 μl of apoptotic thymi were delivered by oropharyngeal aspiration for a total of 10 × 10^6^ cells per mouse. Sixty minutes after instillation of apoptotic cells, mice were sacrificed by intraperitoneal injection of Fatal Plus (2 μl/g of body weight) and tracheotomized. The lungs were lavaged five times with 1 ml PBS. Cells were prepared by cytocentrifugation and stained with a modified Wright-Giemsa stain. To determine the effects of Fabp5-deficiency on the engulfment of apoptotic cells, the efferocytic index was calculated using light microscopy. Ingested cells in a minimum of 400 alveolar macrophages were manually counted per condition. The reader was blinded to the sample identification. The efferocytic index was calculated using the following formula: ((number of ingested apoptotic bodies)/(total macrophages)) × 100. Three independent experiments were performed with 4–5 mice in each group.

### Lipid droplet and lipid uptake assays

Lipid droplets were measured using BODIPY 493/503 (item number 25892; Cayman Chemical). Alveolar macrophages were plated in Dulbecco’s modification of Eagle’s medium containing 10% fetal bovine serum, allowed to adhere, and stained for 30 min in phenol red-free RPMI-based media containing 2 mM L-glutamine, 10 mM glucose, 10% fetal bovine serum, and 760 nM BODIPY. Cells were then washed with HBSS 3 times and fixed with 4% paraformaldehyde. Cells were imaged using a Zeiss LSM 700 confocal microscope according to the manufacturer’s guidelines. Lipid uptake was measured using BODIPY-Palmitate (item number 26749; Cayman Chemical). Alveolar macrophages (100,000 cells/well) were plated in Dulbecco’s modification of Eagle’s medium containing 10% fetal bovine serum, allowed to adhere, washed with HBSS, and starved in serum-free RPMI media containing 2 mM L-glutamine and 10 mM glucose for 1 h. After starvation, cells were washed with HBSS before the addition of 10 ng/ml BODIPY-Palmitate in HBSS. Staining was stopped at a given time point by washing 3 times with HBSS, and cells were fixed with 4% paraformaldehyde. Cells were imaged using a Zeiss LSM 700 confocal microscope according to the manufacturer’s guidelines.

### Metabolomics

Flow-sorted resident alveolar macrophages following the resolution phase of *NTHi* infection were pelleted at 300xg and washed twice before freezing dry pellets at −80°C. Liquid chromatography-mass spectrometry was performed by the Metabolomics core at the University of Colorado Anschutz Medical Campus. Briefly, metabolites were extracted from frozen cell pellets at 2 × 10^6^ cells/ml using cold 5:3:2 methanol:acetonitrile:water via vortexing at 4°C for 30 min followed by centrifugation (10 min, 10,000 g, 4°C) (30). Supernatants (10 uL per injection) were randomized and analyzed on a Thermo Vanquish UHPLC – Thermo Orbitrap Exploris 120 mass spectrometer using a 5 min C18 gradient in positive and negative ion modes in separate runs exactly as described previously (31). Peaks were integrated and metabolites annotated using Maven alongside the KEGG database and an in-house standard library. Quality control was assessed as previously described (32).

### Fatty acid oxidation assay

Fatty acid oxidation was measured using the Fatty Acid Oxidation assay kit (Promega). BAL cells (100,00 cells/well) were plated in Dulbecco’s modification of Eagle’s medium containing 10% fetal bovine serum with or without 20 μM etomoxir. Fatty acid oxidation was measured according to the manufacturer's guidelines using a luminescence Molecular Devices plate reader (SpectraMax iD3).

### Mitochondrial mass and mitochondrial oxidation

Abundance of mitochondria and prevalence of superoxide radicals were measured using MitoLite Blue FX490 (Cayman Chemical; 25159), HSP60 (Cell Signaling Technology; D307), and MitoSox Red (Thermo Fisher Scientific, M36008). Alveolar macrophages (100,00 cells/well) were plated in Dulbecco’s modification of Eagle’s medium containing 10% fetal bovine serum, allowed to adhere, and washed with HBSS. Cells were stained using 100 nM MitoSox and 1X MitoLite for 30 min in HBSS. For HSP60 staining, cells were fixed with 4% paraformaldehyde for 15 min and permeabilized for 15 min using 0.1% Triton X-100. Cells were then blocked for 45 min using 2% bovine serum albumin (BSA). HSP60 antibody (1:50) was added to cells in 0.1% BSA and incubated overnight at 4°C in a humid chamber. Next day, cells were incubated with the secondary antibody (1:500; Thermo Fisher Scientific; 35563) for 1 h. Cells were imaged using a Zeiss LSM 700 confocal microscope according to the manufacturer’s guidelines.

### Microscopy images quantification

Signal intensity of microscopy images was quantified using Fiji 2.16.0. Mean Fluorescence Intensity (MFI) of at least 10 cells was taken across 3 separate experiments for each timepoint and genotype.

### Fluorescence lifetime imaging microscopy (FLIM)

FLIM was performed to detect metabolic changes in alveolar macrophages 0- or 9-days post LPS sterile inflammation using a Zeiss 780 laser-scanning confocal/multiphoton-excitation fluorescence microscope equipped with an ISS A320 FastLIM box and titanium:sapphire MaiTai two-photon laser (Spectra Physics) as previously described (33, 34). Fluorescence of nicotinamide adenine dinucleotide (NADH) and flavin adenine dinucleotide (FAD) were detected simultaneously. To assess mitochondrial activity (OXPHOS), FLIM based optical redox ratio (FLIRR) was calculated as the ratio of bound to enzyme NADH fraction over bound to enzyme FAD fraction. The glycolytic index was calculated as the ratio between free NADH fraction and bound to enzyme NADH fraction.

### Metabolic phenotyping

Oxygen consumption rate (OCR) was measured using the Agilent Seahorse XFe96 Bioanalyzer. Alveolar macrophages (5 × 10^4^ per well) were plated in quadruplets onto Seahorse 96-well plates and pre-incubated in Seahorse XF media (DMEM, 10 mM glucose, 1 mM sodium pyruvate, 2 mM glutamine, pH 7.4) at 37°C for 1 h in the absence of CO_2_. OCR was measured under basal conditions and after sequential addition of Oligomycin, Carbonyl cyanide 4-(trifluoromethoxy)phenylhydrazone (FCCP) and Rotenone/Antimycin A following manufacturer’s instructions. Each measured value was reported on Wave software (Agilent Technologies) and normalized to the number of cells in each well. The cell count per well was determined by fluorescent cell counting using BioTek Cytation one-fifth instrument.

### Lactate assay

Lactate production was measured using the Glycolysis Cell-Based Assay Kit (item number 600450; Cayman Chemical). Cells were plated in phenol-red free RPMI including 1% fetal bovine serum, with or without 2 mM L-glutamine and 10 mM glucose and exposed to 10 pg/ml LPS for 2 h. Lactate production was measured in the supernatant according to the manufacturer’s guidelines.

### U-^13^C-palmitate tracing

Murine bone marrow-derived macrophages (BMDMs) were isolated and differentiated from WT or Fabp5^−/−^ mice as previously described (21). BMDMs were plated at 1 × 10^6^ and treated with 10 μM U-^13^C-Palmitate (Sigma-Aldrich 2483736-17-8) for 6 h before being washed and pelleted at 300xg. Dry pellets were snap-frozen and stored at −80°C. Metabolites from cell pellets were extracted at 2 million cells per ml of cold 5:3:2 methanol:acetonitrile:water via vortexing for 30 min at 4°C. Supernatants were clarified by centrifugation (18,000 *g*, 10 min, 4°C) then transferred to autosampler vials. A pooled QC sample was prepared using equal volume aliquots of each sample. Metabolite extracts were analyzed on a Thermo Vanquish UHPLC coupled to a Thermo Orbitrap Exploris 120 mass spectrometer – 10 uL injections in positive and negative ion modes (separate runs). Metabolites were separated on a Waters Acquity BEH C18 column (2.1 × 100 mm, 1.7 um) at 450 μl/min at 45°C using a 5 min gradient. The negative polarity gradient utilized mobile phases: A = water, 10 mM ammonium acetate; B = 50% acetonitrile, 50% methanol, 10 mM ammonium acetate. The positive polarity gradient utilized mobile phases A = 0.1% formic acid in water, B = 0.1% formic acid in acetonitrile. The gradient program for both polarities was 0–0.5 min 0% B, 0.5–1.1 min 0%–100% B, 1.1–2.75 min hold at 100% B, 2.75–3 min 100%-0% B, 3–5 min hold at 0% B. Eluate was introduced via electrospray ionization (spray voltage: 3 kV for negative polarity, 3.4 kV for positive polarity; sheath gas 50; auxiliary gas 10). The mass spectrometer operated in MS1 mode scanning 65–975 m/z at 120,000 resolution. Instrument stability was assessed via quality control injections of pooled sample aliquots at beginning, end, and throughout the sequence as described (30). Spectra for metabolites and their isotopologues were annotated and integrated using El-Maven (Elucidata); results were graphed using GraphPad Prism (35).

### Assay for transposase-accessible chromatin by sequencing (ATAC-seq) and computational analysis

Flow-sorted resident and recruited WT or Fabp5^−/−^ macrophages were washed twice with 1X PBS prior to counting. Approximately 50,000 cells were pelleted and processed in duplicate for Omni-ATAC-seq as described previously (36). Uniquely indexed libraries were pooled and sequenced on an Illumina NovaSeq 6000 using 150 bp paired-end reads at the Genomics Shared Resource at the University of Anschutz Medical Campus. ATAC-seq reads were trimmed for adapters, length and quality using the bbduk tool from the BBMap Suite (v. 38.05) with arguments ‘ref = adapters.fa ktrim = r qtrim = 10 k = 23 mink = 11 hdist = 1 ftr = 36 maq = 10 minlen = 20’. Quality control was monitored both pre- and post-trim for all samples using FastQC (v. 0.11.8). Trimmed reads were mapped to the mouse genome (mm10; downloaded from http://igenomes.illumina.com.s3-website-us-east-1.amazonaws.com/Mus_musculus/UCSC/mm10/Mus_musculus_UCSC_mm10.tar.gz on December 21, 2021, with corresponding hisat2 index files) using hisat2 (v. 2.1.0) in ‘--very-sensitive’ mode. Resulting SAM files were converted to sorted BAM files using samtools (v. 1.3.1) and then passed to MarkDuplicates (Picard v. 2.6.0) with setting ‘REMOVE_DUPLICATES = true’ to remove PCR and sequencing duplicates. Deduplicated BAM files were converted to bedGraph format and sorted using genomeCoverageBed and sortBed, respectively, from the BEDTools suite (v. 2.25.0). Read coverage was normalized to reads per million mapped using a custom python script and normalized bedGraphs were converted to TDF format using toTDF from igvtools (v. 2.3.75) for visualization in the Integrative Genomics Viewer (IGV). Peak calling was performed using MACS2 (v. 2.1.1) callpeak with ‘--SPMR -f BAMPE -q 0.00001’ arguments on sorted deduplicated BAM files for each pair of replicates, after which ENCODE-blacklisted regions (downloaded from https://github.com/Boyle-Lab/Blacklist/blob/master/lists/mm10-blacklist.v2.bed.gz on December 20, 2021) were removed using bedtools intersect ‘-v’ to yield clean peak files. Clean peak files were subjected to Transcription Factor Enrichment Analysis (TFEA (v. 1.1.4; https://github.com/Dowell-Lab/TFEA; (37)) to detect differential central enrichment of consensus binding motifs represented in the HOCOMOCOv11 core mouse database (downloaded from https://hocomoco11.autosome.ru/final_bundle/hocomoco11/core/MOUSE/mono/HOCOMOCOv11_core_MOUSE_mono_meme_format.meme on December 24, 2021; mapped with a *P*-value cutoff of 1e-5), as described in detail in (38). Enrichment scores (E-scores) for each motif were calculated and corrected for sequence content to reduce known biases associated with local GC enrichment and *P*-values were determined using Z-scores.

### Lung histopathology

Lungs were fixed in 10% phosphate-buffered formalin, dehydrated, embedded in paraffin, and cut at 4 μm thickness. Hematoxylin and eosin-stained lung sections were evaluated in a double-blinded fashion under the light microscope using a modified histopathologic inflammatory scoring system as described previously (39). A final score per mouse on a scale of 0–10 (least to most severe) was obtained based on an assessment of the quantity and quality of peribronchiolar and peribronchial inflammatory infiltrates, luminal exudates and perivascular infiltrates.

### Statistics

Data are expressed as mean ± SEM. Student's *t* test was used for two groups comparison. Differences were considered statistically significant when *P* < 0.05.

## Results

### Alveolar macrophage gene expression is altered in FABP5-deficient mice

**Fig. 1:**
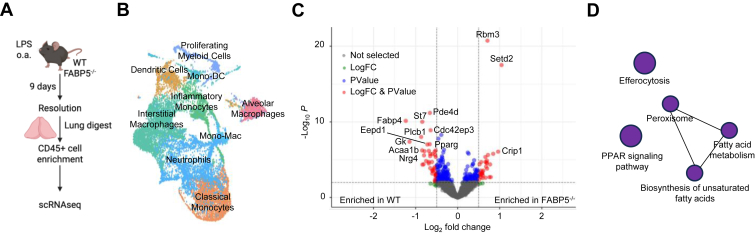
Genetic depletion of Fabp5 alters alveolar macrophages gene expression. WT and Fabp5^−/−^ mice were treated with 15 μg of LPS by oropharyngeal aspiration and sacrificed 9 days later as an inflammation resolution timepoint. A: Schematic of experimental design. B: Single cell RNA-seq UMAP with Seurat clustering of single cell lung suspensions in Loupe Browser 5.0 annotated to define lung myeloid cells. C: Volcano plot of differentially expressed genes (DEGs) in alveolar macrophages between WT and Fabp5^−/−^ mice. *Fabp5* was removed to allow better visualization of other DEGs. Criteria for statistical significance included log_2_ fold change (LogFC) > 0.5 and padj < 0.05 (*P*-value); red dots indicate genes meeting both these criteria, as shown in legend. D: Differentially regulated networks suggested by KEGG pathway analysis database of the downregulated genes in FABP5^−/−^ alveolar macrophages compared to WT.

We previously reported reduced Fatty Acid Binding Protein 5 (FABP5) expression in lung tissue of patients with COPD, especially among patients who reported exacerbations, as well as in a pre-clinical cigarette smoke exposure mouse model (12). To identify whether Fabp5-deletion alters alveolar macrophage gene expression, we exposed WT and Fabp5^−/−^ mice to LPS sterile inflammation (Fig. 1A). Lung tissues were harvested for single-cell RNA sequencing (Fig. 1B) 9 days post-LPS sterile inflammation, a timepoint at which we consistently observe resolution of inflammation as illustrated by weight loss recovery (Supplemental Fig. S1). Differentially expressed genes (DEGs) in alveolar macrophages are presented as a volcano plot where *Fabp5* has been omitted to allow better visualization of other DEGs (Fig. 1C). Pathway analysis of downregulated genes from Fabp5^−/−^ alveolar macrophages compared to WT identified key functional and metabolic pathways of alveolar macrophages, including PPAR signaling, efferocytosis and fatty acid metabolism (Fig. 1D). These data support the hypothesis that FABP5 expression in alveolar macrophages controls both their function and metabolism.

### Alveolar macrophage efferocytic function is decreased in FABP5-deficient mice

**Fig. 2:**
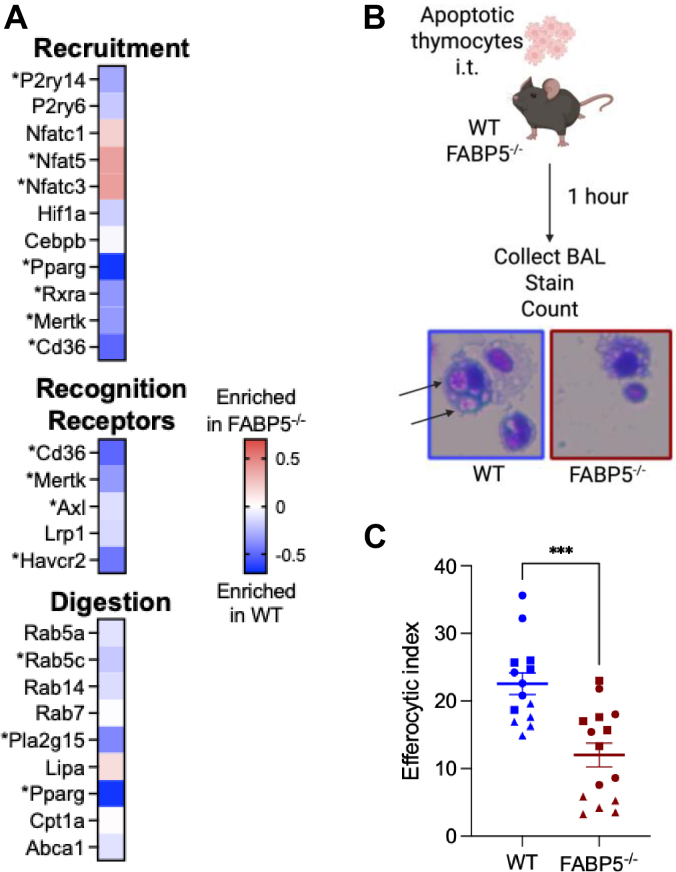
Genetic depletion of Fabp5 in alveolar macrophages reduced efferocytic function. A: Heatmap of differentially expressed efferocytosis genes between WT and Fabp5^−/−^ alveolar macrophages from scRNAseq in Figure 1. Negative values (blue) are enriched in WT, positive values (red) are enriched in Fabp5^−/−^ cells. ∗*P* < 0.05. B: Schematic of experimental design for Fig. 2 C. C: WT (blue) and FABP5^−/−^ (red) mice were instilled intratracheally with apoptotic thymocytes. An hour later, the efferocytic index (ratio of number of apoptotic bodies by total macrophages × 100) was calculated from cytospins of the BAL fluid. ∗∗∗*P* < 0.001. Data are pooled from 3 independent experiments with n = 4–5 mice/genotype. Each experiment is represented with a different symbol.

Given the reduced efferocytosis signaling pathway in Fabp5^−/−^ alveolar macrophages, we compared gene expression between WT and Fabp5^−/−^ cells and identified several genes related to efferocytosis, including recruitment, recognition receptors and digestion, to be downregulated in Fabp5^−/−^ alveolar macrophages (Fig. 2A). To validate whether Fabp5-deficiency impairs efferocytosis in vivo, apoptotic thymocytes were instilled intratracheally in WT and Fabp5^−/−^ mice. Sixty minutes following instillation, the efferocytic index (ratio of number of ingested apoptotic bodies per total macrophages × 100) was calculated from cytospins of the BAL fluid (Fig. 2B). A significant decrease in efferocytosis was observed in Fabp5^−/−^ mice (Fig. 2C) suggesting that in addition to decreased efferocytosis gene expression, the engulfing capabilities of apoptotic cells by Fabp5^−/−^ alveolar macrophages are impaired.

### Reduced lipid uptake and fatty acid oxidation in FABP5-deficient alveolar macrophages

**Fig. 3:**
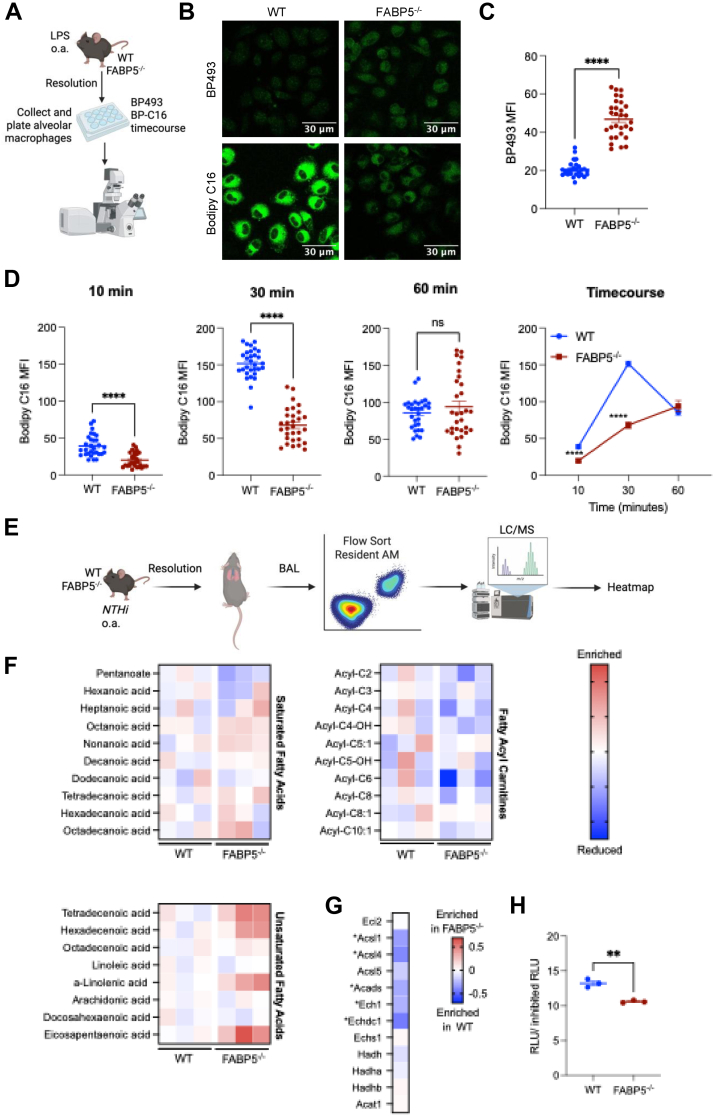
Genetic depletion of Fabp5 in alveolar macrophages reduced fatty acid uptake and oxidation. A: Schematic of experimental design for Figs. 3 B–D. B: Lipid droplets and fatty acid uptake in alveolar macrophages collected 9 days post-LPS sterile inflammation and imaged via confocal microscopy utilizing Bodipy 493 (Top) and Bodipy C16 (Bottom) from WT or Fabp5^−/−^ mice at 10 and 30 min, respectively. Bar represents 30 μm. C: Quantification of lipid droplets measured at 10 min by mean fluorescence intensity (MFI) of Bodipy 493. ∗∗∗∗*P* < 0.0001. D: Quantification of fatty acid uptake assessed at 10, 30, and 60 min. Average mean fluorescence intensity (MFI) at each time point was plotted for each genotype as a time course for Bodipy C16. ∗∗∗∗*P* < 0.0001. E: Schematic of experimental design for Fig. 3 F. F: Heatmaps of saturated and unsaturated fatty acids as well as fatty acyl carnitines assessed by LC-MS from WT and Fabp5^−/−^ flow sorted alveolar macrophages 3 days post-*NTHi* infection. G: Heatmap of differentially expressed fatty acid metabolism enzyme genes between WT and Fabp5^−/−^ alveolar macrophages from scRNAseq. Negative values (blue) are enriched in WT, positive values (red) are enriched in Fabp5^−/−^ cells. H: Fatty acid oxidation quantified via a luminescence-based assay in WT and Fabp5^−/−^ alveolar macrophages 9 days post-LPS sterile inflammation. Data were normalized to the average luminescence observed in samples inhibited with 20 μM etomoxir. ∗∗*P* < 0.01.

Efferocytosis has been shown to promote pro-resolving signaling in macrophages and is coupled with changes in metabolism (40). To test whether the impaired efferocytic function of Fabp5^−/−^ macrophages was associated with impaired fatty acid metabolic regulation, we first examined lipid droplets and fatty acid uptake (Fig. 3A, B). To visualize lipid droplets, we used Bodipy 493/503 (BP493) and measured increased mean fluorescence intensity (MFI) in Fabp5^−/−^ alveolar macrophages compared to WT macrophages during the resolution phase of LPS sterile inflammation (Fig. 3C). To evaluate fatty acid uptake, we used Bodipy-Palmitate (Bodipy-C16) and measured significantly increased Bodipy-C16 MFI in WT cells at 10 and 30 min, but the 60 min timepoint was similar between WT and Fabp5^−/−^ cells. Interestingly, while the Bodipy-C16 MFI in Fabp5^−/−^ cells continued to rise between 10 and 60 min, the Bodipy-C16 MFI in WT cells was maximal at 30 min and started declining at 60 min (Fig. 3D). Similar observations were obtained in naïve WT alveolar macrophages (Supplemental Fig. S2). Taken together, these data suggest that WT cells may be incorporating and degrading the fatty acid derivative Bodipy-C16 faster than Fabp5^−/−^ cells.

Next, we explored fatty acid metabolism by comparing short and long chain fatty acid abundance in flow-sorted WT or Fabp5^−/−^ alveolar macrophages during the resolution phase post-*NTHi* infection by liquid chromatography coupled with mass spectrometry (LC-MS) (Fig. 3E). We reasoned that impaired fatty acid oxidation could be inferred from increased abundance of long chain fatty acid relative to short chain intracellular fatty acid content. Indeed, we detected higher levels of long chain fatty acids coupled with lower levels of short chain fatty acids in Fabp5^−/−^ alveolar macrophages when compared to WT cells (Fig. 3F). Several enzymes involved in fatty acid metabolism were downregulated in Fabp5^−/−^ alveolar macrophages compared to WT in cells isolated from lung tissues during the resolution phase post LPS sterile inflammation (Fig. 3G). We confirmed those findings by directly measuring reduced fatty acid oxidation in Fabp5^−/−^ alveolar macrophages compared to WT collected during the resolution phase of LPS sterile inflammation (Fig. 3H). These results suggest that Fabp5 is essential for normal fatty acid uptake and fatty acid oxidation in alveolar macrophages.

### Reduced mitochondrial mass and oxygen consumption rate in Fabp5-deficient alveolar macrophages

**Fig. 4:**
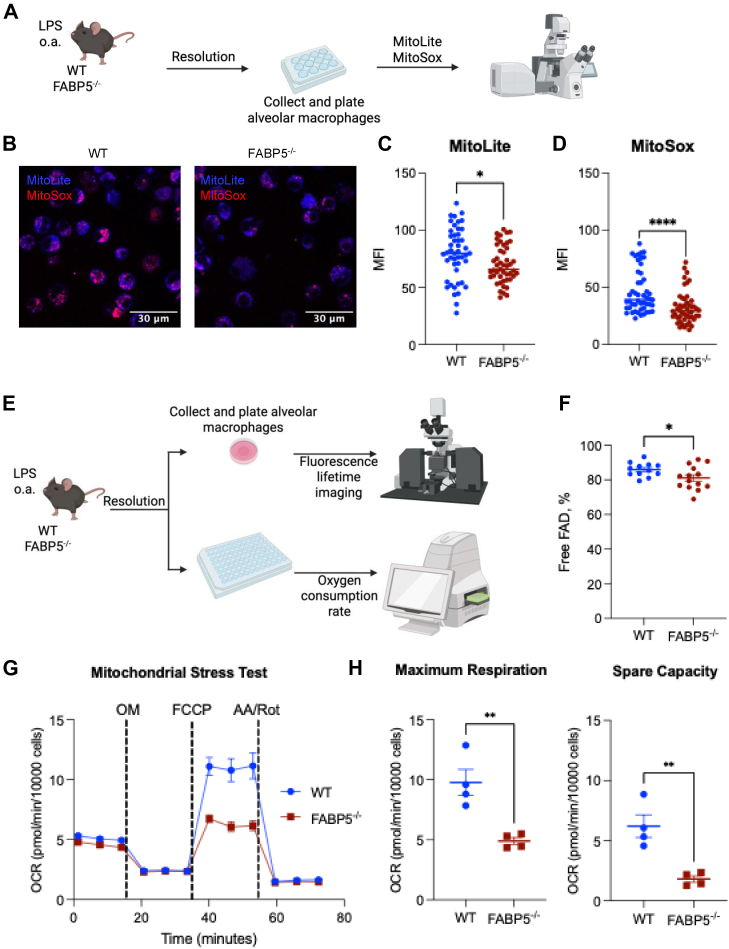
Genetic depletion of Fabp5 in alveolar macrophages reduces mitochondrial mass and oxygen consumption rate. A: Schematic of experimental design for Figs. 4 B–D. B: MitoLite (blue) and MitoSox (red) images of WT and Fabp5^−/−^ alveolar macrophages 9 days post- LPS sterile inflammation. Bar represents 30 μm. C: Quantification of mitochondrial mass assessed using average mean fluorescence intensity (MFI) of MitoLite. n = 50/genotype. ∗*P* < 0.05. D: Quantification of mitochondrial superoxide production using average mean fluorescence intensity (MFI) of MitoSox. n = 50/genotype. ∗∗∗*P* < 0.0001. E: Schematic of experimental design for Figs. 4 F–H. F: Oxidative phosphorylation measured as percent free FAD by fluorescent lifetime imaging microscopy. G: Oxygen Consumption Rate (OCR) measurements of WT (blue) and Fabp5^−/−^ (red) alveolar macrophages 9 days post-LPS sterile inflammation. OM = oligomycin A; FCCP = Carbonyl cyanide 4-(trifluoromethoxy)phenylhydrazone; AA = antimycin A; Rot = Rotenone. H: Fundamental parameters of oxidative metabolism including maximum respiration, and spare respiratory capacity in WT (blue) and FABP5^−/−^ (red) alveolar macrophages 9 days post-LPS sterile inflammation. ∗∗*P* < 0.01. Data are representative of three independent experiments.

To determine whether mitochondrial function is altered in the absence of Fabp5 in alveolar macrophages, we labeled alveolar macrophages collected during the resolution phase of LPS sterile inflammation with MitoLite and Mitosox (Fig. 4A, B). We measured reduced mitochondrial mass in Fabp5^−/−^ compared to WT alveolar macrophages as illustrated by reduced MitoLite mean fluorescence intensity (MFI) (Fig. 4C). MitoSox MFI, used to determine mitochondrial oxidation, was significantly lower in Fabp5^−/−^ alveolar macrophages compared to WT (Fig. 4D). To confirm the reduction in mitochondrial mass, we also stained naïve WT and Fabp5^−/−^ alveolar macrophages for MitoLite, MitoSox, and Hsp60 for its mitochondrial localization. All these markers were significantly reduced in naïve Fabp5^−/−^ alveolar macrophages compared to WT (Supplemental Fig. S3). Additionally, we assessed oxidative phosphorylation metabolic capacity in alveolar macrophages using the Seahorse XF Cell Mito Stress Test on WT and Fabp5^−/−^ alveolar macrophages collected during the resolution phase of LPS sterile inflammation (Fig. 4E). As illustrated in Figure 4F, and consistent with our previously published data in bone marrow-derived macrophages (BMDM) (21), Fabp5^−/−^ alveolar macrophages displayed a sharp reduction of their mitochondrial oxidative capabilities in comparison to WT cells, shown by a large decline of the maximal respiration and spare respiratory capacity (Fig. 4G). These data suggest that mitochondrial dysfunction or a shift in energy metabolism in Fabp5-deficient alveolar macrophages may contribute to their reduced efferocytic function relative to WT cells.

### Accumulation of TCA cycle metabolites and glycolysis intermediaries in Fabp5-deficient alveolar macrophages

**Fig. 5:**
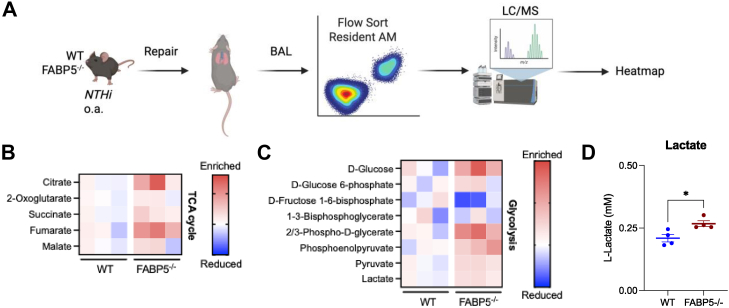
Genetic depletion of Fabp5 in alveolar macrophages increased accumulation of TCA and glycolysis metabolites. A: Schematic of experimental design for Figs. 5 B–C. B: Heatmaps of TCA metabolites assessed from flow-sorted resident alveolar macrophages 3 days post-*NTHi* infection by LC-MS. n = 3 samples/genotype. C: Heatmaps of glycolysis metabolites assessed from flow sorted resident alveolar macrophages 3 days post-*NTHi* infection by LC-MS. n = 3 samples/genotype. D: Secretion of L-lactate by alveolar macrophages cultured for 2 h following LPS stimulation. n = 4 samples/genotype. ∗*P* < 0.05.

The tricarboxylic acid (TCA) cycle oxidizes substrates to generate NADH and FADH_2_, which are required to generate ATP by the electron transport chain through oxidative phosphorylation. This ATP generation has been suggested to be essential for efferocytosis and the energy required to reorganize the actin cytoskeleton (41). Using flow-sorted resident BAL macrophages isolated from WT or Fabp5^−/−^ mice in the resolution phase post-*NTHi* infection, we examined TCA cycle and glycolysis metabolites using LC-MS (Fig. 5A). We measured increased levels of TCA metabolites, including citrate, succinate, and fumarate, in Fabp5^−/−^ compared to WT macrophages (Fig. 5B). Interestingly, pro-inflammatory murine macrophages have been shown to present disrupted TCA cycle flux at steps mediated by isocitrate dehydrogenase and succinate dehydrogenase (42). Similarly, glycolysis metabolites were increased in Fabp5^−/−^ resident BAL macrophages compared to WT macrophages (Fig. 5C). Accumulation of TCA and glycolysis metabolites was also detected in naïve alveolar macrophages (Supplemental Fig. S4). We confirmed a significant increase in lactate secretion by Fabp5^−/−^ alveolar macrophages compared to WT when cells were plated for 2 h on cell culture plates (Fig. 5D). These data suggest that Fabp5^−/−^ alveolar macrophages have an altered TCA cycle and may favor glycolysis as their source of energy at baseline and during the resolution post-infection or post LPS sterile inflammation, which is consistent with the failure of Fabp5^−/−^ macrophages in transitioning towards a pro-resolving programming.

### Altered TCA cycle and reduced metabolic activity in Fabp5-deficient bone marrow-derived macrophages

**Fig. 6:**
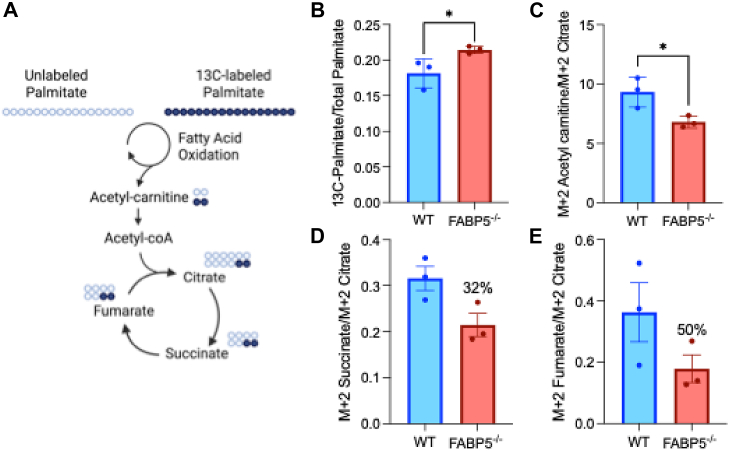
Altered TCA cycle and reduced metabolic activity in Fabp5-deficient bone marrow-derived macrophages. A: Schematic representation of palmitate tracing and incorporation of 13-C labelled carbons into TCA cycle metabolites. B: Fraction of M+2 palmitate over total Palmitate (labeled and unlabeled). C: Fractional enrichment of Palmitate-derived M+2 acetyl carnitine relative to M+2 citrate. D: Fractional enrichment of Palmitate-derived M+2 succinate relative to M+2 citrate. E: Fractional enrichment of Palmitate-derived M+2 fumarate relative to M+2 citrate. n = 3 samples/genotype. ∗*P* < 0.05.

While metabolomics measures the abundance of metabolites and can reveal metabolic changes dictated by increased or decreased substrate usage, it does not discriminate between the two. Hence, to assess the ability of Fabp5^−/−^ macrophages to perform mitochondrial fatty acid oxidation and downstream TCA cycle propagation, we performed stable isotope tracing using (U-13C) palmitic acid to determine differences in metabolic flux resulting from fatty acid oxidation (Fig. 6A). We measured increased incorporation of labeled palmitate in Fabp5^−/−^ macrophages indicating that palmitate metabolism is reduced in the absence of Fabp5 (Fig. 6B). Additionally, we measured a significant reduction in the acetyl carnitine to citrate ratio in Fabp5^−/−^ macrophages signaling a deficiency in energy production where acetyl-CoA derived from palmitate is not being efficiently converted into acetyl-carnitine or utilized in the TCA cycle confirming our findings of lower fatty acid oxidation by Fabp5^−/−^ alveolar macrophages (Fig. 6C). We also measured decreased ratios of succinate to citrate (Fig. 6D) and fumarate to citrate (Fig. 6E) in Fabp5^−/−^ macrophages confirming an alteration of the TCA cycle that may reflect a shift from oxidative metabolism towards alternative metabolic pathways or reduced metabolic activity in Fabp5^−/−^ macrophages.

### AP-1 transcription factor family motifs are enriched in regions with increased chromatin accessibility in Fabp5-deficient alveolar macrophages

**Fig. 7:**
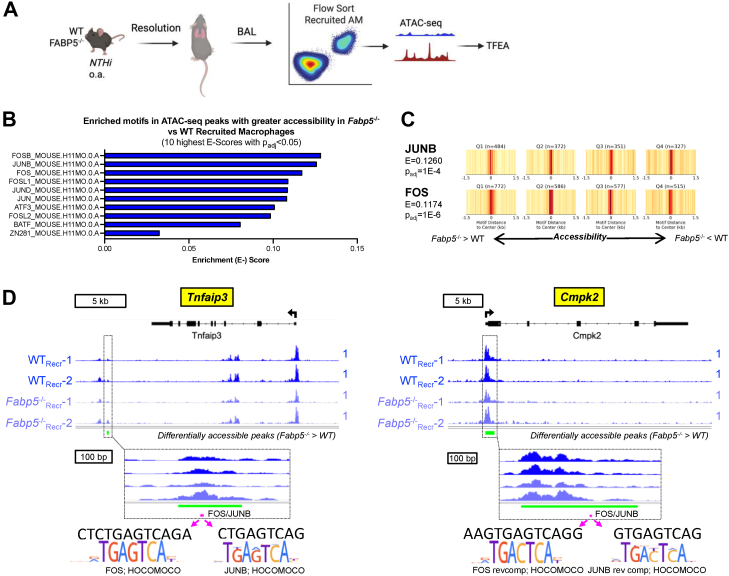
Enrichment for AP-1 family binding motifs in regions exhibiting greater chromatin accessibility in Fabp5^−/−^ versus WT recruited alveolar macrophages. A: Schematic of experimental design. B: Enrichment (E−) Scores output by TFEA of the 10 most significantly enriched transcription factor binding motifs in ATAC-seq peaks with greater accessibility in Fabp5^−/−^ versus WT recruited macrophages 3 days post-*NTHi* infection. C: FOS and JUNB motif displacement distributions from TFEA of differentially regulated MACS2-called ATAC-seq peaks. Each column represents the frequency of the indicated motif instance at the specified distance from the ATAC-seq peak center (labeled 0), where color specifies a normalized frequency relative to the entire 3-kb region. Darker colors indicate greater enrichment on a 0 to 1 scale. D: Representative examples of IGV-visualized ATAC-seq tracks showing peaks with significantly greater accessibility in Fabp5^−/−^ versus WT recruited macrophages (highlighted by green boxes) that contain matches to FOS/JUNB binding motifs (indicated by pink boxes). ATAC-seq, assay for transposase-accessible chromatin using sequencing; TFEA, transcription factor enrichment analysis; E-Score, enrichment score; P-adj, adjusted *P*-value; IGV, Integrative Genomics V.iewer.

To further interrogate the underlying programming of Fabp5^−/−^ macrophages during the resolution of inflammation, we performed genome-wide profiling of chromatin accessibility using the Assay for Transposase-Accessible Chromatin using sequencing (ATAC-seq) in flow-sorted recruited macrophages isolated from WT and Fabp5^−/−^ mice during the resolution phase of *NTHi* infection. Following ATAC-seq, we used a Transcription Factor Enrichment Analysis (TFEA) pipeline (43) to probe differential central enrichment for consensus transcription factor binding motifs in the differentially accessible ATAC-seq peak sequences identified in Fabp5^−/−^ versus WT macrophages (Fig. 7A). This analysis identified significant central enrichment for AP-1 transcription factors binding motifs, including FOSB, JUNB, FOS, FOSL1, JUND and JUN, within regions exhibiting greater accessibility in Fabp5^−/−^ versus WT recruited BAL macrophages (Fig. 7B, C). Examples of loci that show increased FOS/JUNB accessibility in Fabp5^−/−^ versus WT recruited macrophages are shown in Fig. 7D & Supplemental Fig. S5. Interestingly, in both examples, the increased ATAC-seq peak accessibility in regions harboring FOS/JUNB motifs in Fabp5^−/−^ versus WT recruited macrophages was associated with a general decrease in accessibility in the nearest gene bodies *Tnfaip3*, *Cmpk2*, and *Fabp5*, often indicative of transcriptional repression. Tnfaip3 is involved in reducing inflammation (44), and Cmpk2 is a mitochondrial gene that controls macrophage homeostasis (45). Additionally, we examined the degree of lung inflammation still present 3 days post-NTHi infection. While WT mouse lung inflammation was fully resolved, Fabp5^−/−^ mouse lung remained slightly inflamed (Supplemental Fig. S6). These findings are consistent with Fabp5^−/−^ recruited macrophages exhibiting delayed resolution compared to WT recruited macrophages in response to *NTHi*-induced lung inflammation.

## Discussion

Our results indicate that Fabp5 plays a critical role in macrophage function and metabolism during the resolution of inflammation. Our findings support a model in which reduced Fabp5 expression contributes to delayed pro-resolving programming of lung macrophages with impaired fatty acid metabolism, mitochondrial function and efferocytosis.

Akin to the initiation of inflammation, the resolution of inflammation is a highly regulated and active process. If left unchecked, inflammation drives excessive tissue damage. Therefore, clearance of dead bodies and inflammatory cells, as well as activation of repair mechanisms by mononuclear phagocytes, are required for the resolution of infection-induced inflammation. The engulfment of apoptotic cells, known as efferocytosis, causes vast changes in macrophage metabolism that are thought to promote pro-resolving signaling, which is key to the resolution of inflammation (40). Efferocytosis is actually an essential part of tissue homeostasis (46) and if apoptotic cells are not effectively cleared, they may contribute to tissue inflammation and chronic inflammatory diseases (47, 48). In our previous study (16), we showed that Fabp5^−/−^ mice had persistent inflammation following influenza infection. Here we show impaired efferocytosis by Fabp5^−/−^ alveolar macrophages that was accompanied by defects in fatty acid uptake and metabolism as well as reduced mitochondrial function. It has been demonstrated that FABP5 is a potent activator of immunosuppressive macrophage function, particularly in the context of tumor-associated macrophages (49, 50). We also previously described increased pro-inflammatory programming of Fabp5^−/−^ bone marrow-derived macrophages (21), consistent with Fabp5 playing an important general role in the resolution of inflammation.

Interestingly, resident macrophages are imprinted by the tissue in which they reside. Although they may be replaced by circulating monocytes following infection, the lung tissues instruct their specific transcriptional or metabolic program required for tissue homeostasis (51, 52, 53). Alveolar macrophages are derived from fetal liver monocytes that differentiate into macrophages following exposure to granulocyte-macrophage colony-stimulating factor (GM-CSF) produced by alveolar type II epithelial cells (54). In the absence of GM-CSF, alveolar macrophage development is impaired (55) leading to alveolar proteinosis (56). PPARγ expression is increased by GM-CSF binding to its receptor on fetal monocytes (57), which leads to the expression of genes involved in lipid metabolism, storage, and degradation. We recently demonstrated a crosstalk between FABP5 and PPARγ in which (i) FABP5 and PPARγ physically interact with one another, (ii) FABP5 expression increases PPARγ activity, and (iii) in turn, PPARγ increases FABP5 expression through direct transcriptional modulation (21). Additionally, it has been suggested that the differentiation of monocytes into alveolar macrophages is associated with the loss of AP-1 activity (58). Here, our ATAC-seq-TFEA analysis indicated increased AP-1 activity in Fabp5^−/−^ alveolar macrophages which suggests a defect in differentiation and proper alveolar macrophage pro-repair licensing. Additionally, the apparent reduction in accessibility across *Tnfaip3* and *Cmpk2* gene bodies in Fabp5^−/−^ recruited macrophages compared to WT suggests not only that a critical negative feedback regulator of inflammatory signaling (44), but also macrophage homeostasis and control of inflammation (45), may not be fully operational in the absence of Fabp5.

Lung macrophage numbers are increased in COPD BAL (59) and are thought to contribute to COPD pathogenesis. At the same time, phagocytic capacities of alveolar macrophages, and efferocytosis in particular, have been shown to be impaired in COPD (5, 60). Several metabolic alterations have been described in COPD alveolar macrophages, including a decrease in maximal respiration and spare respiratory capacity (61). It is now well accepted that macrophage metabolism has a profound impact on their function, and reciprocally, their functions affect their metabolic phenotypes (42, 62). For instance, a pro-inflammatory macrophage has been associated with glycolysis, whereas oxidative phosphorylation is associated with a reparative macrophage (63). Fatty acid oxidation serves as an important input to the TCA cycle, generating Acetyl-CoA, which feeds into oxaloacetate. Increased fatty acid oxidation and incorporation into the TCA metabolite pool has been implicated as an important function in pro-resolution reprogramming in macrophages (64). In this study, we show that Fabp5^−/−^ alveolar macrophages had increased glycolysis and TCA metabolites compared to WT during the repair phase. Using labelled palmitate, we measured decreased ratios of succinate to citrate and fumarate to citrate in Fabp5^−/−^ bone marrow-derived macrophages confirming an alteration of the TCA cycle in the absence of Fabp5.

The alveolar space is a particularly unique metabolic niche characterized by low glucose and high oxygen levels, which has led to the understanding that alveolar macrophages do not rely on glycolysis during LPS-induced inflammation (65). This is demonstrated by the fact that while Fabp5^−/−^ alveolar macrophages produce higher levels of extracellular lactate, the total concentration is still much lower than the levels produced by blood monocyte-derived macrophages (66). It has been shown that alveolar macrophages can adopt a glycolytic phenotype in response to hypoxia, a hallmark of severe COPD, restoring pro-inflammatory cytokine production (67, 68). In this report, we used two different models of lung inflammation: a sterile inflammation (LPS) and an infection-driven inflammation (*NTHi*). Although the two models present different severity and kinetics, they both converged to show that Fabp5 deficiency led to dysregulated lung macrophage metabolism that may contribute to the persistence of an inflammatory response. Since we have previously shown that FABP5 expression is decreased in COPD, we speculate that Fabp5 depletion leaves alveolar macrophages more susceptible to metabolic reprogramming resulting in a more robust pro-inflammatory phenotype. Our results also show that Fabp5 concomitantly increases fatty acid metabolism, as shown by the strong differences in free fatty acid content, the lower fatty acid uptake and fatty acid oxidation in the absence of Fabp5 during resolution of inflammation. Additionally, we measured reduced maximal respiration and spare respiratory capacity in Fabp5^−/−^ alveolar macrophages, similar to what was illustrated in COPD alveolar macrophages (61). Hence, metabolic alterations in FABP5-deficient macrophages may disrupt pro-resolving macrophage programming in COPD, contributing to disease progression.

Overall, our data suggest FABP5 as a target to rewire alveolar macrophage metabolism and programming towards a pro-resolving phenotype to reduce inflammation in COPD without compromising overall host immune responses.

## Data availability

Data will be shared upon request to Dr Gally (National Jewish Health, gallyf@njhealth.org). Sequencing data will be deposited in a repository after acceptance of the manuscript.

## Supplemental data

This article contains supplemental data.

## Conflict of interest

The authors declare that they have no conflicts of interest with the contents of this article.
