# Supplementary material for: Fatty acid-binding protein 5 deficiency impairs alveolar macrophage function and metabolism
Source: J Lipid Res. 2026 Jun 23;67(7):101069. doi: 10.1016/j.jlr.2026.101069 (PMC13320021; doi:10.1016/j.jlr.2026.101069)
Supplement: Supplementary File [file mmc1.docx]

**SUPPLEMENTAL FIGURES**

**
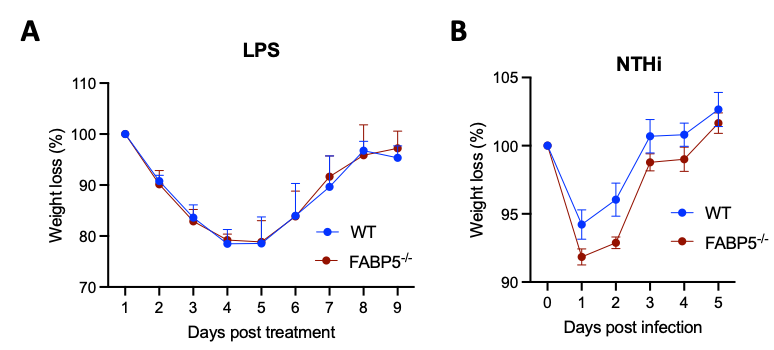
**

**Figure E1: Weight loss following LPS sterile inflammation or *NTHi* infection. A.** WT and FABP5^-/-^ mice were treated with 20 µg LPS by oropharyngeal aspiration and weight loss was measured every day. n = 7-9 mice/group. **B.** WT and FABP5^-/-^ mice were infected with 2.5 x 10^5^ *NTHi* CFUs and weight loss was measured every day. n = 9-10 mice/group.


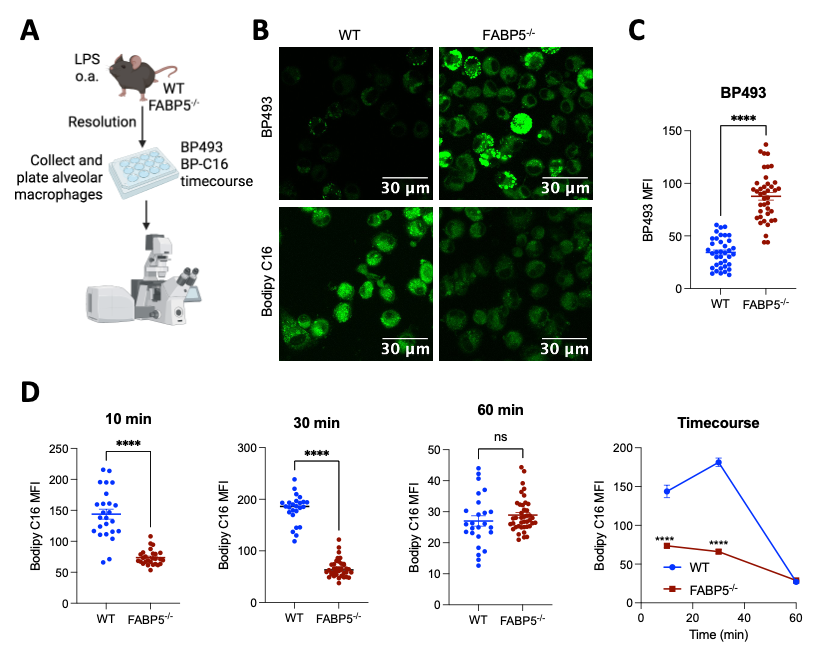


**Figure E2**: **Genetic depletion of FABP5 in naïve alveolar macrophages increases fatty acid droplets and decreases fatty acid uptake. A.** Schematic of experimental design **B.** Lipid droplets and fatty acid uptake in alveolar macrophages imaged via confocal microscopy utilizing Bodipy 493 (Top) and Bodipy C16 (Bottom) at 30 minutes from WT or FABP5^-/-^ mice. Bar represents 30 µm. **C.** Quantification of lipid droplets measured by mean fluorescence intensity (MFI) of Bodipy 493. ****p < 0.0001. **D.** Quantification of fatty acid uptake assessed at 10, 30, and 60 minutes. Average mean fluorescence intensity (MFI) at each time point was plotted for each genotype as a time course for Bodipy C16. ****p < 0.0001.


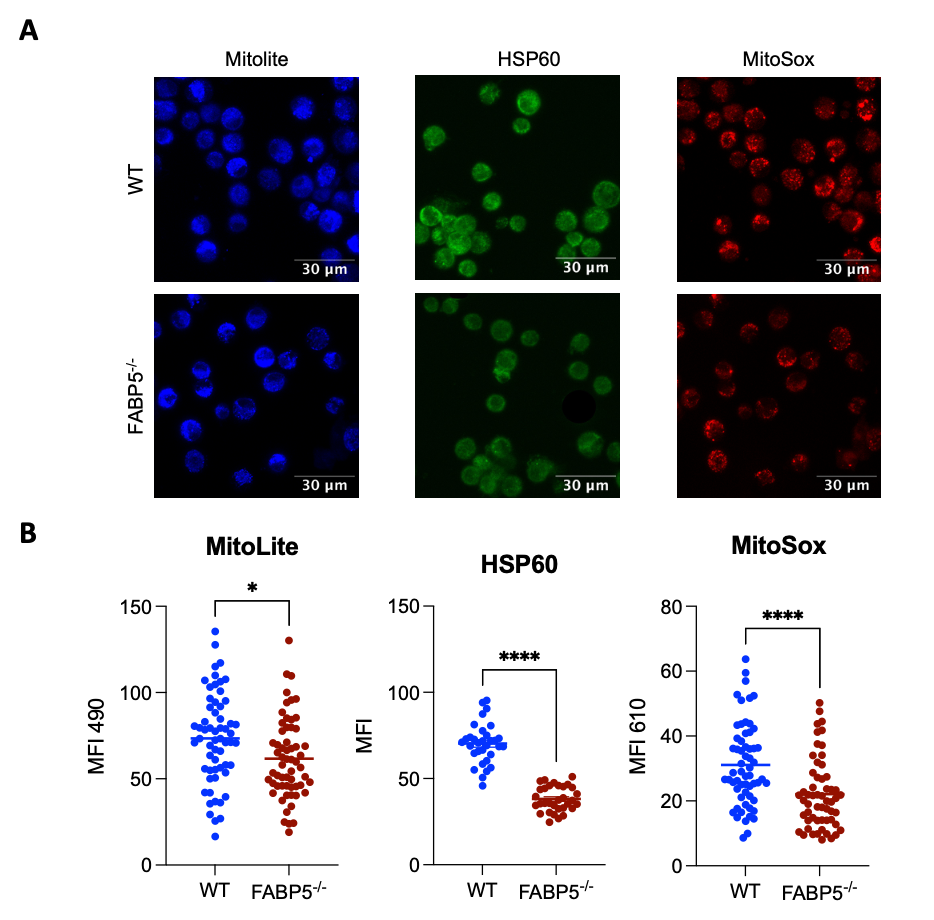


**Figure E3**: **Genetic depletion of FABP5 in naïve alveolar macrophages decreases mitochondrial mass and superoxide production. A.** MitoLite (blue), HSP60 (green), and MitoSox (red) images in WT or FABP5^-/-^ naïve alveolar macrophages. Bar represents 30 µm. **B.** Quantification of mitochondrial mass assessed using average mean fluorescence intensity (MFI) of MitoLite and HSP60 as well as quantification of mitochondrial superoxide production using average mean fluorescence intensity (MFI) of MitoSox. n = 30-60/genotype. *p < 0.05 ****p < 0.0001.


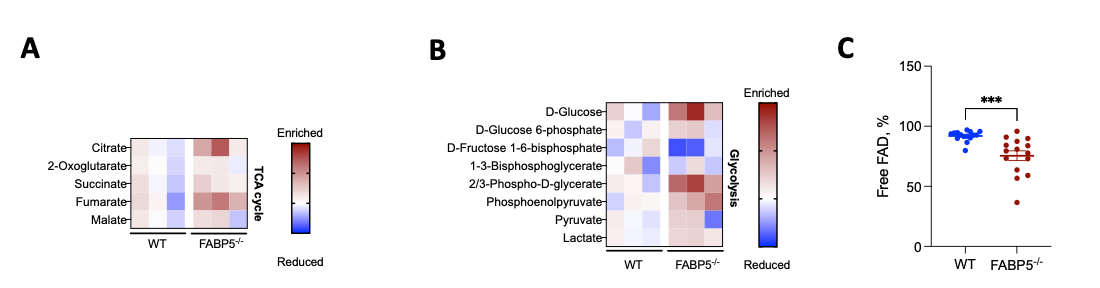


**Figure E4**: **Genetic depletion of FABP5 in naïve alveolar macrophages increases TCA and glycolysis metabolites while decreasing oxidative phosphorylation. A.** Heatmaps of TCA metabolites assessed from WT or FABP5^-/-^ naïve alveolar macrophages by LC-MS. n=3 samples/genotype. **B.** Heatmaps of TCA metabolites assessed from WT or FABP5^-/-^ naïve alveolar macrophages by LC-MS. n=3 samples/genotype. **C.** Percent free FAD measured by lifetime imaging from WT or FABP5^-/-^ naïve alveolar macrophages. **** p < 0.0001.


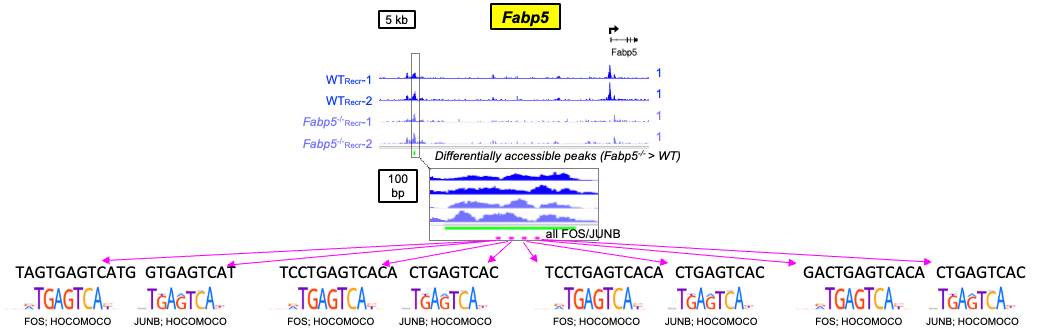


**Figure E5**: **Genetic depletion of FABP5 in recruited alveolar macrophages increases AP-1 transcription factors enrichment at the *Fabp5* locus.** IGV-visualized ATAC-seq tracks with MACS2-called peaks (*boxed* in *green* and magnified below panel) at the *Fabp5* gene locus exhibiting an increased FOS/JUNB occupancy in FABP5^-/-^ recruited macrophages compared to WT. ATAC-seq, assay for transposase-accessible chromatin using sequencing; IGV, Integrative Genomics Viewer.


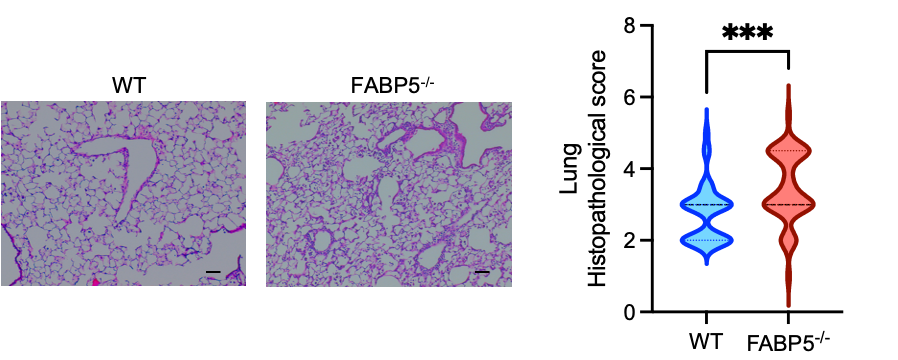


**Figure E6**: **Increased inflammation in FABP5^-/-^ mouse lung tissues 3 days post *NTHi* infection. A.** H&E staining of WT and FABP5^-/-^ mouse lung tissues. Bar represents 100 µm. **B.** Quantification of lung histopathology based on H&E staining. **** p < 0.001.
